# Quantifying the risk of drug-associated skin hyperpigmentation relative to the general population: A national cohort study using TriNetX

**DOI:** 10.1016/j.jdin.2025.08.011

**Published:** 2025-09-29

**Authors:** Debby Cheng, Helen Ji, David Xiang, Christopher J. Thang, Aurore D. Zhang, Arash Mostaghimi, Yevgeniy R. Semenov, Nicholas Theodosakis

**Affiliations:** a Harvard Medical School, Boston, Massachusetts; b Department of Dermatology, Massachusetts General Hospital, Boston, Massachusetts; c Department of Dermatology, Brigham and Women’s Hospital, Boston, Massachusetts

**Keywords:** adverse drug reactions, amitriptyline, carbamazepine, cohort study, doxycycline, drug-induced hyperpigmentation, epidemiology, hydrochlorothiazide, hyperpigmentation, medication-induced pigmentation, melanin accumulation, minocycline, phenytoin, propensity score matching, retrospective analysis, skin discoloration, TriNetX

*To the Editor:* While multiple drugs have been linked to hyperpigmentation, data quantifying conferred risk remain limited.^1^ This study quantifies background prevalence of hyperpigmentation in the general population and estimates the additional risk associated with 6 medications commonly implicated in drug-induced hyperpigmentation (DIH): doxycycline, minocycline, phenytoin, carbamazepine, amitriptyline, and hydrochlorothiazide. Analyses were conducted using TriNetX (105 health care organizations; Supplementary Methods, available via Mendeley at https://data.mendeley.com/datasets/scsbz22jmt/1).

To establish baseline rates of hyperpigmentation, we conducted a cross-sectional analysis of patients with ≥2 billing codes for general medical examinations, excluding individuals prescribed any of the study drugs. Between 2024-2025, the 1-year incidence of hyperpigmentation was 1.1%, and the prevalence was 7.7%, with incidence peaking at ages 65-69 (Supplementary Tables, available via Mendeley at https://data.mendeley.com/datasets/scsbz22jmt/1).

To assess DIH risk, we identified adults without prior hyperpigmentation in 6 treatment cohorts: (1) acne treated with doxycycline or (2) minocycline, (3) seizures treated with phenytoin or (4) carbamazepine, (5) depression treated with amitriptyline, and (6) hypertension treated with hydrochlorothiazide. Each cohort was compared to 2 control groups receiving alternative medications. 1:1 propensity score matching accounted for demographics, socioeconomic factors, and disease severity (Supplementary Tables I-IV, available via Mendeley at https://data.mendeley.com/datasets/scsbz22jmt/1). Risk ratios and 95% confidence intervals were calculated at 1-year (Table I), 3-year (Table II), and 5-year (Supplementary Tables, available via Mendeley at https://data.mendeley.com/datasets/scsbz22jmt/1) intervals post-index (drug initiation).

**Table I:** Risk of skin hyperpigmentation at 1-year follow-up in patients on study drugs, compared to propensity score-matched controls

Drug	Control	Treatment group	Control group	Risk ratio (95% CI)
		Number of eligible individuals (No. of outcomes, 1-y)	Number of eligible individuals (No. of outcomes, 1-y)	
Doxycycline	Cephalexin	3004 (137)	3054 (62)	**2.25**∗**(1.67, 3.02)**
Doxycycline	Erythromycin	4616 (209)	4874 (80)	**2.76**∗**(2.14, 3.56)**
Minocycline	Cephalexin	3017 (131)	2932 (54)	**2.36**∗**(1.72, 3.22)**
Minocycline	Erythromycin	4450 (176)	4483 (75)	**2.36**∗**(1.81, 3.09)**
Phenytoin	Valproate	23,141 (54)	23,067 (46)	1.17 (0.79, 1.73)
Phenytoin	Levetiracetam	15,855 (47)	15,738 (46)	1.01 (0.68, 1.52)
Carbamazepine	Valproate	14,104 (58)	14,086 (28)	**2.07**∗**(1.32, 3.25)**
Carbamazepine	Levetiracetam	11,195 (43)	11,122 (35)	1.22 (0.78, .191)
Amitriptyline	Duloxetine	19,135 (105)	19,105 (136)	0.77 (0.60, 1.00)
Amitriptyline	Sertraline	17,712 (94)	17,703 (109)	0.86 (0.66, 1.14)
Hydrochlorothiazide	Amlodipine	645,452 (4521)	643,611 (4031)	**1.12**∗**(1.07, 1.17)**
Hydrochlorothiazide	Lisinopril	614,847 (4332)	614,653 (3841)	**1.13**∗**(1.08, 1.18)**

∗ Statistically significant at *P* < .05.

**Table II:** Risk of skin hyperpigmentation at 3-year follow-up in patients on study drugs, compared to propensity score-matched controls

Drug	Control	Treatment group	Control group	Risk ratio (95% CI)
		Number of eligible individuals (No. of outcomes, 3-y)	Number of eligible individuals (No. of outcomes, 3-y)	
Doxycycline	Cephalexin	3004 (222)	3054 (137)	**1.65**∗**(1.34, 2.03)**
Doxycycline	Erythromycin	4616 (369)	4874 (182)	**2.14**∗**(1.80, 2.55)**
Minocycline	Cephalexin	3017 (194)	2932 (125)	**1.51**∗**(1.21, 1.88)**
Minocycline	Erythromycin	4450 (259)	4483 (163)	**1.60**∗**(1.32, 1.94)**
Phenytoin	Valproate	23,141 (152)	23,067 (130)	1.17 (0.92, 1.47)
Phenytoin	Levetiracetam	15,855 (102)	15,738 (117)	0.87 (0.66, 1.13)
Carbamazepine	Valproate	14,104 (139)	14,086 (80)	**1.74**∗**(1.32, 2.28)**
Carbamazepine	Levetiracetam	11,195 (107)	11,122 (89)	1.19 (0.90, 1.58)
Amitriptyline	Duloxetine	19,135 (267)	19,105 (303)	0.88 (0.75, 1.04)
Amitriptyline	Sertraline	17,712 (239)	17,703 (248)	0.96 (0.81, 1.15)
Hydrochlorothiazide	Amlodipine	645,452 (11,217)	643,611 (9569)	**1.17**∗**(1.14, 1.20)**
Hydrochlorothiazide	Lisinopril	614,847 (10,868)	614,653 (9453)	**1.15**∗**(1.12, 1.18)**

∗ Statistically significant at *P* < .05.

Doxycycline was associated with increased hyperpigmentation risk vs control medications at 1 (risk ratio_Control_ [95% confidence interval] = 2.76_Erythromycin_ [2.14-3.56]; 2.25_Cephalexin_ [1.67-3.02]), 3 (2.14_Erythromycin_ [1.80-2.55]; 1.65_Cephalexin_ [1.34-2.03]), and 5 years (1.89_Erythromycin_ [1.63-2.19]; 1.61_Cephalexin_ [1.34-1.94]). Minocycline showed increased risk at 1 (2.36_Erythromycin_ [1.81-3.09]; 2.36_Cephalexin_ [1.72-3.22]), 3 (1.60_Erythromycin_ [1.32-1.94]; 1.51_Cephalexin_ [1.21-1.88]), and 5 years (1.43_Erythromycin_ [1.21-1.69]; 1.41_Cephalexin_ [1.16-1.72]). Carbamazepine was associated with increased risk at 1 (2.07 [1.32-3.25]), 3 (1.74 [1.32-2.28]), and 5 years (2.01 [1.59-2.53]) compared to valproate, but only at 5 years compared to levetiracetam (1.28 [1.02-1.60]). Phenytoin was associated with increased risk only at 5 years (1.24_Valproate_ [1.02-1.52]). Amitriptyline showed no significant association. Hydrochlorothiazide was associated with increased risk at 1 (1.12_Amlodipine_ [1.07-1.17]; 1.13_Lisinopril_ [1.08-1.18]), 3 (1.17_Amlodipine_ [1.14-1.20]; 1.15_Lisinopril_ [1.12-1.18]), and 5 years (1.26_Amlodipine_ [1.23-1.29]; 1.17_Lisinopril_ [1.14-1.19]).

These findings suggest that while approximately 8% of the general population has clinically significant hyperpigmentation, certain medications confer modest but measurable increased risk. Notably, within 1 year of drug initiation, doxycycline and minocycline were associated with more than twofold increased risk, hydrochlorothiazide with a 12% to 13% increased risk, while phenytoin and amitriptyline showed no significant association.

While minocycline is a more well-documented cause of DIH,^2^ our results suggest doxycycline may confer comparable or slightly higher risk, consistent with a recent retrospective study.^3^ The progressive increase in risk over time for doxycycline, minocycline, carbamazepine, and hydrochlorothiazide supports prior observations that DIH may develop gradually over months to years.^2^

This study offers key epidemiologic data to inform patient counseling and treatment decisions. Limitations include inability to assess medication dosage, adherence, and misdiagnoses, mitigated by requiring ≥2 billing codes for all diagnoses and medications, and using previously validated algorithms for identifying hyperpigmentation.^4^ Future research should explore how DIH risk varies with medication dosage, sun exposure, and polypharmacy.

## Conflicts of interest

Dr Mostaghimi reports receiving honorarium from: hims and hers, AbbVie, Sun Pharma, Pfizer, Digital Diagnostics, Lilly, Equillium, ASLAN, Boehringer Ingelheim, Figure 1, Dermatheory, Olaplex, Legacy Healthcare, and Pelage. Dr Semenov is an advisory board member/consultant and has received honoraria from Incyte Corporation, Castle Biosciences, Galderma, and Sanofi outside of the submitted work. Authors Cheng, Ji, Xiang, Thang, Zhang, and Dr Theodosakis have no conflicts of interest to declare.
